# Comparative secretome analysis of *Trichoderma asperellum* S4F8 and *Trichoderma reesei* Rut C30 during solid-state fermentation on sugarcane bagasse

**DOI:** 10.1186/1754-6834-6-172

**Published:** 2013-11-29

**Authors:** Isa Jacoba Marx, Niël van Wyk, Salome Smit, Daniel Jacobson, Marinda Viljoen-Bloom, Heinrich Volschenk

**Affiliations:** 1 Department of Microbiology, Stellenbosch University, Private Bag X1, Matieland 7602, Stellenbosch, South Africa; 2 MS Unit, Proteomics Laboratory, Central Analytical Facility, Stellenbosch University, Private Bag X1, Matieland 7602, Stellenbosch, South Africa; 3 Division of Molecular Biology and Human Genetics, Faculty of Medicine and Health Sciences, Stellenbosch University, Francie van Zijl Drive, PO Box 19063, Tygerberg 7505, South Africa; 4 Institute for Wine Biotechnology, Faculty of AgriSciences, Stellenbosch University, Private Bag X1, 7602, Matieland, South Africa

**Keywords:** *Trichoderma asperellum* S4F8, *Trichoderma reesei* Rut C30, Secretome, Solid-state fermentation, Sugarcane bagasse, Proteomics

## Abstract

**Background:**

The lignocellulosic enzymes of *Trichoderma* species have received particular attention with regard to biomass conversion to biofuels, but the production cost of these enzymes remains a significant hurdle for their commercial application. In this study, we quantitatively compared the lignocellulolytic enzyme profile of a newly isolated *Trichoderma asperellum* S4F8 strain with that of *Trichoderma reesei* Rut C30, cultured on sugarcane bagasse (SCB) using solid-state fermentation (SSF).

**Results:**

Comparison of the lignocellulolytic enzyme profiles of S4F8 and Rut C30 showed that S4F8 had significantly higher hemicellulase and β-glucosidase enzyme activities. Liquid chromatography tandem mass spectrometry analysis of the two fungal secretomes enabled the detection of 815 proteins in total, with 418 and 397 proteins being specific for S4F8 and Rut C30, respectively, and 174 proteins being common to both strains. In-depth analysis of the associated biological functions and the representation of glycoside hydrolase family members within the two secretomes indicated that the S4F8 secretome contained a higher diversity of main and side chain hemicellulases and β-glucosidases, and an increased abundance of some of these proteins compared with the Rut C30 secretome.

**Conclusions:**

In SCB SSF, *T. asperellum* S4F8 produced a more complex lignocellulolytic cocktail, with enhanced hemicellulose and cellobiose hydrolysis potential, compared with *T. reesei* Rut C30. This bodes well for the development of a more cost-effective and efficient lignocellulolytic enzyme cocktail from *T. asperellum* for lignocellulosic feedstock hydrolysis.

## Background

Lignocellulases (cellulases, hemicellulases, and ligninases) are the key enzymes involved in lignocellulose depolymerization, and have a wide array of industrial applications. Perhaps the most promising is their application in the bioconversion of lignocellulosic plant material to fermentable monomeric sugars, an essential step in second-generation bioethanol production [1]. Although significant progress has been made in the enzymatic saccharification of lignocellulosic feedstocks [2], full commercial-scale implementation is hampered by a number of factors, including the high cost of the enzymes required for efficient lignocellulose hydrolysis. One of the contributing factors is the intrinsic recalcitrance of plant cell walls, which demand high enzyme loadings for efficient degradation [3]. Multi-faceted approaches to reduce enzyme production costs and/or improve the efficiency of enzyme cocktails have therefore received growing attention, and a number of approaches are in use, including streamlining of bioprocess designs, development of cheaper feedstocks for enzyme production, improving and designing feedstock-specific cellulase cocktails, and bioengineering microorganisms expressing lignocellulolytic enzymes [4].

Solid-state fermentation (SSF), the culturing of microorganisms on moist solid substrates in order to mimic their natural physiology and growth environment, is an age-old, but resurgent culturing method for the production of lignocellulolytic enzymes [5-9]. The technical and economic benefits of SSF over traditional submerged fermentation include superior volumetric enzyme productivity, simpler fermenter design and downstream processing, lower aeration demands, no agitation requirements, lower sterility demands (due to lower water activity) and lower effluent generation [8,9]. Furthermore, SSF offers a biological process to convert cheap, under-utilized agro-industrial wastes (either as carbon/energy source or as an inert carrier) into high-value end products such as organic acids, flavour and aroma compounds, secondary metabolites, and industrially relevant enzymes [6].

The production of cellulases and hemicellulases via SSF has been investigated using different substrates and microorganisms [5,8]. The choice of appropriate substrate is important for the successful production of fungal enzymes, as complex feedstocks are known to induce expression of complex lignocellulolytic enzyme cocktails to ensure complete substrate hydrolysis [10]. Several *Trichoderma* species have been successfully cultivated on various lignocellulosic substrates under SSF conditions, and their important enzymes characterized, including cellulases from the *T. reesei* Rut C30 strain (hereafter referred to as Rut C30) [11-14].

Sugarcane bagasse (SCB), one of the world’s most abundant agricultural wastes, has been utilized in SSF systems for a variety of applications [15]. These include culturing of bacteria, yeasts, and filamentous fungi for the production of citric acid and various glycoside hydrolases, including endoglucanases, β-glucosidases, α-amylases, and xylanases [16-19]. Following extraction of the sugar from the cane, the remaining fibrous material (bagasse), containing approximately 40–50% cellulose, 25–35% hemicellulose, 7–29% lignin and less than 4% ash, serves as an ideal substrate for growth and induction of lignocellulolytic enzymes [17-20].

Because of their high secretion capacity and relatively high specific enzyme activities, several *Trichoderma* species [21] and their inexpensive cultivation via SSF on various agricultural waste products to produce lignocellulases have previously been investigated [22-24]. Secretome studies to identify and quantify the major cellulases, hemicellulases, and accessory enzymes involved in the depolymerization and degradation of agricultural waste products have also been conducted [25,26]. Quantitative approaches to investigate the secretome of Rut C30 identified 350 secretory proteins, with the large majority being associated with cellulolytic and proteolytic enzymes [27]. A complementary study later identified 636 proteins secreted by *T. reesei*, of which 354 were quantified [28]. Although *T. reesei* is currently the main industrial source of commercial cellulases, it has a relatively poor repertoire of cellulases compared with other fungi [29]. The lack of potent hemicellulases and the low levels of β-glucosidase and other accessory enzymes in the secretome of industrially important *T. reesei* strains have prompted investigations into other fungal strains and/or enzymes that could potentially replace and/or supplement the *T. reesei* cellulases [30].

In the present study, the lignocellulolytic isolate S4F8, identified as a *Trichoderma asperellum* strain, was characterized in terms of its cellulase and hemicellulase enzymes when cultivated on untreated SCB in a simulated SSF process. The enzyme characteristics of *T. asperellum* S4F8 (hereafter referred to as S4F8) were compared with those of the benchmark Rut C30 strain, and comparative secretome analysis was used to differentiate between the enzyme cocktails produced by the two fungal strains.

## Results and discussion

### Isolation and identification of fungal isolate S4F8

During an extensive screen for culturable lignocellulolytic soil fungi, S4F8 outperformed other isolates with regard to growth on synthetic (carboxymethylcellulose (CMC), hydroxyethylcellulose (HEC), Avicel, and beechwood xylan (BWX)) and natural (wheat bran, triticale bran, and SCB) lignocellulosic substrates as the sole carbon source (data not shown). These results suggested that S4F8 most likely produces a well-balanced repertoire of core and accessory lignocellulosic enzymes required to degrade these substrates, and was therefore chosen for further enzyme characterization.

The 594 bp internal transcribed spacer (ITS) sequence amplified from isolate S4F8 displayed 100% homology to the partial ITS 1 and 2 regions of an uncultured Hypocreales clone [Genbank EF086981.1]. Five out of five conserved anchors (oligonucleotide barcodes) for the genus *Hypocrea* were identified in S4F8, using the TrichOKey barcode system [31], which is widely used for the identification of *Trichoderma* species originating from different geographical locations [31,32]. The S4F8 ITS sequence also showed 100% sequence identity to 40 *T. asperellum* species in the TrichoBLAST database, and was therefore identified as a *T. asperellum* strain belonging to the XII Rufa clade, section *Pachybasium* ‘A’.

Strains of *T. asperellum*, which are frequently isolated from soil, plant roots and tissues, fungal biomass, and dead wood, have mostly been studied as mycoparasitic fungi with application as biocontrol agents [33,34]. In contrast to studies on Rut C30, studies on the extracellular hydrolytic enzymes of *T. asperellum* have been limited to the identification of proteins linked to its antagonistic interactions with other fungi and plants [35-41]. Further investigation was therefore required to characterize the lignocellulolytic enzymes expressed by *T. asperellum* strain S4F8.

### Optimization of SSF culture conditions

Given the established success of Rut C30 in SSF, this culturing system was selected for a comparative study of the hydrolytic enzymes produced by S4F8 and Rut C30. An initial screening under different SSF conditions indicated that the highest enzyme activities of endoxylanase, β-xylosidase, endoglucanase, cellobiohydrolase I and β-glucosidase for both S4F8 and Rut C30 were recorded after 3 days of incubation on SCB (data not shown) as opposed to the 7 days typically reported for fungal SCB SSF [10,42].

The myriad of different conditions reported for *Trichoderma* SSF does not allow a proper comparison of the enzyme levels and activities for the different systems. Mekala and co-workers reported up to 25.6 filter paper units (FPU) per gram of dry substrate (gds) for Rut C30 in SCB SSF, whereas *Trichoderma harzianum* produced 12.8 U/ml xylanase on 280 g/l substrate after 7 days of incubation [43]. The latter study indicated that several experimental parameters influenced enzyme yields, including incubation time, extraction methods, and substrate loading. Other factors that improved cellulase production by *T. reesei* during SSF included relative humidity and temperature [14], continuous light exposure [44], aeration and higher substrate concentrations [6].

In the present study, S4F8 yielded marginally higher endoglucanase and β-xylosidase activities when incubated in darkness under controlled relative humidity (RH) of 90% (culture condition C) compared with the standard culture condition A (30°C in darkness without RH control), whereas exposure to light (culture condition B) had a generally negative effect on the enzyme activities of endoxylanase and side chain hemicellulases (Table 1). Because none of the modifications to the standard SSF conditions significantly improved the important enzyme activities, the standard conditions were used in subsequent experiments.

**Table 1:** **Enzyme activity profiles for *T. asperellum *** **S4F8 cultured under different SCB SSF conditions**

**SSF culture conditions (3 days)**	**Enzyme activity (U/gds)**						
	**Cellulases**			**Main chain hemicellulases**		**Side chain hemicellulases**	
	**Endoglucanase**	**Cellobiohydrolase I**	**β-Glucosidase**	**Endoxylanase**	**β-Xylosidase**	**α-Galactosidase**	**α-Arabinofuranosidase**
A: 30°C, without light (standard conditions)	1.40 ±0.07^a^	0.32 ±0.03	1.01 ±0.25	14.84 ±0.81	4.78 ±0.31	1.16 ±0.14	1.31 ±0.08^e^
B: 30°C with light	1.40 ±0.26	0.28 ±0.07	0.72 ±0.25	10.92 ±2.45	4.46 ±0.37	0.90 ±0.08	0.99 ±0.07
C: standard conditions plus RH of 90%	1.95 ±0.30^b^	0.34 ±0.03	0.97 ±0.23	14.61 ±1.99	5.78 ±0.42^b^	0.99 ±0.15	1.22 ±0.03^e^
D: 26°C, without light	1.50 ±0.05	0.36 ±0.05	1.24 ±0.44	13.22 ±2.00	3.68 ±0.26^c^	1.38 ±0.24^d^	1.06 ±0.05

gds, Gram of dry substrate; RH, relative humidity; SSF, solid-state fermentation.

^a^Mean value of triplicate experiments (n = 3), with standard deviations indicated for each mean value.

Significant difference (*P* < 0.03) compared with ^b^A, B, and D; ^c^A, B, and C; ^d^B and C; and ^e^B and D.

### Characterization of lignocellulolytic enzyme activities produced during SCB SSF

**Figure 1:**
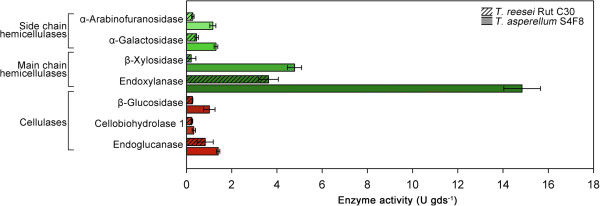
**Comparison of cellulase (red) and hemicellulase (green) activities in sugarcane bagasse (SCB) solid-state fermentation (SSF) extracts produced by *Trichoderma reesei *Rut C30 (striped bars) and *Trichoderma asperellum *S4F8 (solid bars).** Filtered SSF extracts from *T. reesei* Rut C30 and *T. asperellum* S4F8 cultured in triplicate under standard SCB SSF conditions for 3 days were subjected to enzyme activity analysis. Endoglucanase and endoxylanase activities were measured by dinitrosalicyclic acid (DNS) assay, while β-glucosidase, cellobiohydrolase I, α-arabinofuranosidase, β-xylosidase, and α-galactosidase activities were determined with the respective *p*-nitrophenyl substrates. Error bars denote standard deviations from the mean values of triplicate measurements (n = 3).

Hemicellulose, like lignin, acts as a physical barrier that protects cellulose against enzymatic degradation, but this barrier can be overcome through the synergistic action of enzyme cocktails with enhanced hemicellulolytic capabilities [45]. The present study found that the S4F8 SSF extract contained particularly high levels of main chain hemicellulases, endoxylanase (14.8 U/gds) and β-xylosidase (4.7/U gds), with a 4-fold and 23-fold higher activity, respectively, compared with that of Rut C30 (Figure 1). In terms of side chain hemicellulase enzyme activities, the S4F8 SSF extract furthermore displayed three-fold to four-fold higher levels of α-arabinofuranosidase and α-galactosidase activity. Compared with Rut C30, S4F8 showed comparable cellobiohydrolase I and endoglucanase activities and a three-fold higher β-glucosidase activity. This enzyme activity profile suggested that culturing S4F8 on untreated SCB using SSF produced an enzyme cocktail with enhanced hemicellulose degradation ability compared with that of Rut C30. As commercial *T. reesei* cellulase preparations are typically low in β-glucosidase activity, supplementation with exogenous β-glucosidases, either by homologous or heterologous expression of β-glucosidase genes, or co-cultivation of *T. reesei* with other high β-glucosidase-producing fungi, is often required for efficient hydrolysis of complex substrates [46]. Consequently, the enhanced β-glucosidase activity of S4F8 could render it suitable to meet this requirement.

### Protein profiling of *T. asperellum* S4F8 and *T. reesei* Rut C30 secretomes

Proteomics has greatly contributed to the current understanding of the enzymes involved in lignocellulosic hydrolysis, and brought us closer to elucidating the complete set of enzymes required for effective hydrolysis of complex substrates. The first proteomic investigations into the secretome of *T. reesei*[25] identified 22 and 36 proteins in strains Rut C30 and CL847, respectively, with the majority of these proteins being linked to cellulose and hemicellulose hydrolysis. More recently, the iTRAQ system has enabled quantitative analysis of the Rut C30 secretome, in which 636 secreted proteins were identified, with 230 proteins (36%) associated with cellulolytic and proteolytic enzymes [28].

A proteomic approach using liquid chromatography tandem mass spectrometry (LC-MS/MS) was used in this study to quantitatively compare the S4F8 and Rut C30 secretomes in a SCB SSF process, using a single time point and temperature. In total, 815 proteins were identified in the SSF extracts, with 418 and 397 proteins being specific to the S4F8 and Rut C30 extracts, respectively, and 174 proteins being common to both species (see Additional file 1: Table S1; see Additional file 2: Table S2). This high number of detected proteins could be attributable to the possibly higher induction of a large subset of enzymes during SCB SSF, and/or the high sensitivity of the LTQ Orbitrap Velos system.

Within the combined S4F8 and Rut C30 secretomes, N-terminal Sec-dependent secretion signals [47] were identified *in silico* for 315 proteins (39% of the total proteins detected), with 180 and 135 secreted proteins being predicted for S4F8 and Rut C30, respectively. The presence of more than 60% of the proteins in the secretomes without predicted secretion signals indicates possible cell lysis, cell death or non-classic secretory mechanisms.

**Figure 2:**
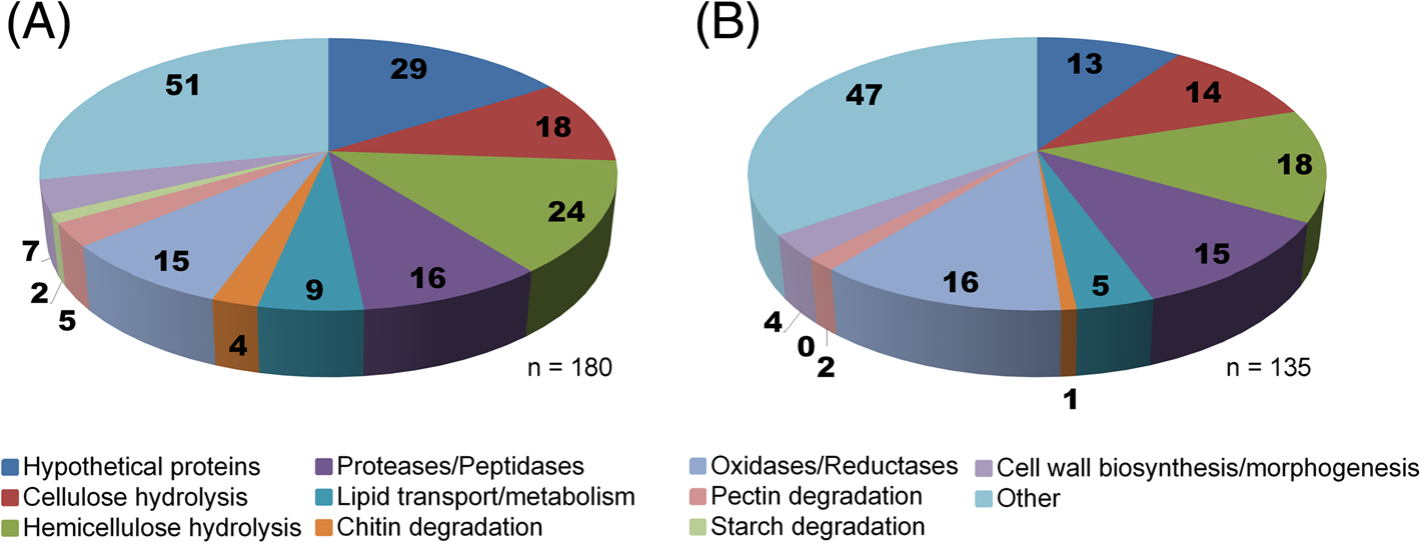
**Grouping of secreted proteins according to biological function for the sugarcane bagasse (SCB) solid-state fermentation (SSF) secretomes of (A) *Trichoderma asperellum *S4F8 and (B) *Trichoderma reesei *Rut C30.** Biological function predictions were based on the Joint Genome Institute (JGI) genome database for *T. asperellum* CBS 433.97 version 1.0 and *T. reesei* RUT C-30 version 1.0.

The predicted secreted proteins were grouped according to their biological function (Figure 2). Within the combined S4F8 and Rut C30 secretomes, 68 proteins (23% of total secreted proteins) were identified (false discovery rate (FDR) ≤1.0) as having either putative esterase (5 proteins) or glycoside hydrolase (63 proteins) activity relevant to lignocellulose degradation. The percentage of proteins acting on cellulose and hemicellulose (relative to the total secreted) was marginally higher in S4F8 (21%) than in Rut C30 (18%). Similarly, a higher number of proteins involved in cellulose and hemicellulose degradation were detected in the S4F8 secretome (18 and 24 proteins, respectively) compared with the Rut C30 secretome (14 and 18 proteins, respectively). Included in the enzyme profile of both S4F8 and Rut C30 were expansin-like proteins such as swollenin (>jgi|Trias1|58369, >jgi|Trias1|57959, >jgi|TrireRUTC30_1|104220), which play a non-hydrolytic role in the disruption of lignocellulose (see Additional file 3: Table S3). This study also identified several substrate binding proteins such as CBM1 cellulose binding domain Cip2 (>jgi|TrireRUTC30_1|125575) and Cip (>jgi|TrireRUTC30_1|121449) in the Rut C30 secretome, and CBM13 (>jgi|Trias1|149192) in the S4F8 secretome. No extracellular lignin-degrading enzymes such as lignin peroxidases, manganese peroxidases, or laccases were detected in the S4F8 and Rut C30 secretomes, including the two recently predicted *T. asperellum* extracellular laccases *sensu stricto*[48]. However, several predicted proteins, including metal-containing oxidases and other oxidoreductases potentially linked to lignin degradation, were detected in the S4F8 (15 proteins) and Rut C30 (16 proteins) secretomes. In addition to the lignocellulolytic-related enzymes, the S4F8 and Rut C30 secretomes contained a set of proteases and peptidases (15 proteins detected in both secretomes), proteins involved in lipid transport and metabolism (9 for S4F8 and 5 for Rut C30), pectin degradation (5 for S4F8 and 2 for Rut C30), chitin degradation (4 for S4F8 and 1 for Rut C30), and cell wall biosynthesis and morphogenesis (7 for S4F8 and 4 for Rut C30), while the S4F8 secretome contained two proteins involved in starch hydrolysis (none was found for Rut C30) (Figure 2).

**Figure 3:**
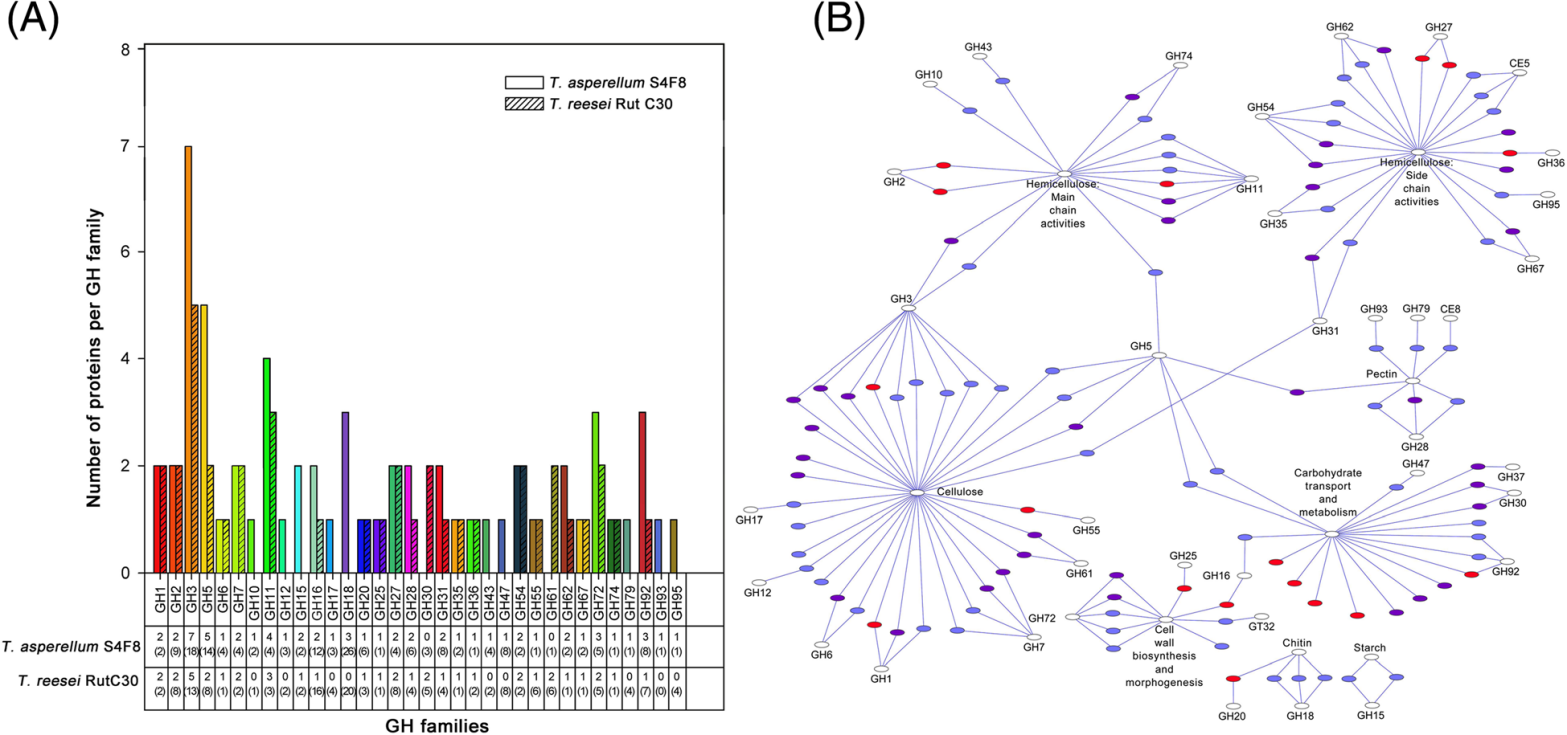
**Grouping and distribution analysis of glycoside hydrolase (GH) and functional network analysis. (A)** Number and distribution of GHs from each GH family detected in the secretomes of *Trichoderma asperellum* S4F8 and *Trichoderma reesei* Rut C30. Numbers in brackets represent the total potential number of GH enzymes per family, based on the annotated genome sequences for *T. asperellum* CBS 433.97 version 1.0 and *T. reesei* RUT C-30 version 1.0. (JGI genome database). **(B)** Functional annotation network analysis of *T. asperellum* S4F8 and *T. reesei* Rut C30 secretomes. Secreted proteins involved in cellulose, hemicellulose, pectin, chitin, starch degradation, cell wall biosynthesis and morphogenesis, and general carbohydrate transport and metabolism are displayed with purple nodes representing *T. reesei* Rut C30, blue nodes representing *T. asperellum* S4F8, and red nodes representing proteins found in both secretomes. For a detailed version of the functional annotation network that includes enzyme identities, see Additional file 4: Figure S1.

Grouping and distribution analysis of the secreted proteins according to glycoside hydrolase (GH) families into 34 different GH families (according to the carbohydrate-active enzyme database, CAZy, http://www.cazy.org) further highlighted the diverse enzymatic profile of the S4F8 and Rut C30 secretomes (Figure 3A, Table 2). Firstly, not all of the predicted GHs (from the respective annotated genome sequence databases) were detected in the S4F8 and Rut C30 secretomes during SCB SSF; 36% of the total (potential) GH proteins were found in the S4F8 secretome, as opposed to 25% representation in the Rut C30 secretome (tabulated summary in Figure 3A). It was noteworthy that all the potential representatives of the GH1 (β-glucosidases), GH11 (endoxylanases), GH25 (*N*,*O*-diacetylmuramidase), GH54 and GH62 (α-L/N-arabinofuranosidases), and GH74 (xyloglucanases) families were detected in both strains.

**Table 2:** **Summary of glycoside hydrolase (GH) family protein representatives detected in the secretomes of *Trichoderma asperellum *** **S4F8 and *Trichoderma reesei *** **Rut C30**

**GH Family**	***T. asperellum *** **S4F8**		***T. reesei *** **Rut C30**	
	**Protein ID**	**Predicted protein**	**Protein ID**	**Predicted protein**
**GH1**	>jgi|Trias1|63798	β-Glucosidase	>jgi|TrireRUTC30_1|127115	β-Glucosidase
	>jgi|Trias1|55643	β-Glucosidase	>jgi|TrireRUTC30_1|77989	β-Glucosidase
**GH2**	>jgi|Trias1|148204	β-Mannosidase	>jgi|TrireRUTC30_1|67432	β-Mannosidase
	>jgi|Trias1|152923	β-Mannosidase	>jgi|TrireRUTC30_1|12549	β-Mannosidase
**GH3**	>jgi|Trias1|128828	β-Glucosidase	>jgi|TrireRUTC30_1|136547	β-Glucosidase
	>jgi|Trias1|151383	β-Glucosidase	>jgi|TrireRUTC30_1|8750	β-Glucosidase
	>jgi|Trias1|203210	β-Glucosidase	>jgi|TrireRUTC30_1|125268	β-Glucosidase
	>jgi|Trias1|23916	β-Glucosidase	>jgi|TrireRUTC30_1|25095	β-Glucosidase
	>jgi|Trias1|63437	β-Glucosidase		
	>jgi|Trias1|65584	β-Glucosidase		
	>jgi|Trias1|62211	β-Xylosidase	>jgi|TrireRUTC30_1|140746	β-Xylosidase
**GH5**	>jgi|Trias1|193120	β-Glucocerebrosidase	>jgi|TrireRUTC30_1|11580	Endo-β-1,6-galactanase
	>jgi|Trias1|194740	β-Glucocerebrosidase		
	>jgi|Trias1|150477	β-Mannase		
	>jgi|Trias1|356270	Endoglucanase 2	>jgi|TrireRUTC30_1|72489	Endoglucanase 2
	>jgi|Trias1|61451	Endoglucanase 2		
**GH6**	>jgi|Trias1|84972	Exoglucanase 2	>jgi|TrireRUTC30_1|122470	Exoglucanase 2
**GH7**	>jgi|Trias1|46985	Exoglucanase 1	>jgi|TrireRUTC30_1|125125	Exoglucanase 1
	>jgi|Trias1|57926	Endoglucanase	>jgi|TrireRUTC30_1|5304	Endoglucanase 1
**GH10**	>jgi|Trias1|53366	Endo-1,4-β-xylanase 3		
**GH11**	>jgi|Trias1|179571	Endo-1,4-β-xylanase 1	>jgi|TrireRUTC30_1|134945	Endo-1,4-β-xylanase 1
	>jgi|Trias1|244563	Endo-1,4-β-xylanase 1	>jgi|TrireRUTC30_1|124931	Endo-1,4-β-xylanase 2
	>jgi|Trias1|83211	Endo-1,4-β-xylanase 1	>jgi|TrireRUTC30_1|38418	Endo-1,4-β-xylanase 1
	>jgi|Trias1|90115	Endo-1,4-β-xylanase 1		
**GH12**	>jgi|Trias1|177701	Endoglucanase 1		
**GH15**	>jgi|Trias1|135222	Glucoamylase		
	>jgi|Trias1|151475	Glucoamylase		
**GH16**	>jgi|Trias1|198977	Transglycosylase	>jgi|TrireRUTC30_1|66752	Glucanosyltransferase
	>jgi|Trias1|97006	Glucanosyltransferase		
**GH17**	>jgi|Trias1|62474	Glucan 1,3-β-Glucosidase		
**GH18**	>jgi|Trias1|148765	Chitinase		
	>jgi|Trias1|57384	Chitinase		
	>jgi|Trias1|41515	Chitinase		
**GH20**	>jgi|Trias1|157721	β-N-acetylhexosaminidase	>jgi|TrireRUTC30_1|99285	β-N-acetylhexosaminidase
**GH25**	>jgi|Trias1|24131	*N*,*O*-diacetylmuramidase	>jgi|TrireRUTC30_1|13308	*N*,*O*-diacetylmuramidase
**GH27**	>jgi|Trias1|59499	α-D-Galactosidase	>jgi|TrireRUTC30_1|6433	α-D-galactosidase
	>jgi|Trias1|47755	α-D-Galactosidase	>jgi|TrireRUTC30_1|71638	α-D-galactosidase
**GH28**	>jgi|Trias1|204961	Endo-polygalacturonase	>jgi|TrireRUTC30_1|133383	Endopolygalacturonase
	>jgi|Trias1|74014	Exo-polygalacturonase		
**GH30**			>jgi|TrireRUTC30_1|90847	β-Glucocerebrosidase
			>jgi|TrireRUTC30_1|93498	β-Glucocerebrosidase
**GH31**	>jgi|Trias1|132074	α-Glucosidase		
	>jgi|Trias1|62176	α-Xylosidase	>jgi|TrireRUTC30_1|134448	α-Xylosidase
**GH35**	>jgi|Trias1|51088	β-Galactosidase	>jgi|TrireRUTC30_1|101346	β-Galactosidase
**GH36**	>jgi|Trias1|179128	α-Galactosidase 2	>jgi|TrireRUTC30_1|12566	α-Galactosidase 2
**GH43**	>jgi|Trias1|56700	Xylosidase		
**GH47**	>jgi|Trias1|233323	α-1,2-Mannosidase		
**GH54**	>jgi|Trias1|152723	α-N-arabinofuranosidase B	>jgi|TrireRUTC30_1|102517	α-L-arabinofuranosidase B
	>jgi|Trias1|55003	α-N-arabinofuranosidase B	>jgi|TrireRUTC30_1|72252	α-L-arabinofuranosidase B
**GH55**	>jgi|Trias1|127630	Glucan 1,3-β-glucosidase	>jgi|TrireRUTC30_1|25104	Glucan 1,3-β-glucosidase
**GH61**			>jgi|TrireRUTC30_1|122518	Endoglucanase 7
			>jgi|TrireRUTC30_1|139633	Endoglucanase 4
**GH62**	>jgi|Trias1|138627	α-L-arabinofuranosidase	>jgi|TrireRUTC30_1|118070	α-N-arabinofuranosidase
	>jgi|Trias1|53918	α-N-arabinofuranosidase		
**GH67**	>jgi|Trias1|328757	α-Glucuronidase	>jgi|TrireRUTC30_1|90302	α-Glucuronidase
**GH72**	>jgi|Trias1|140372	β-1,3-Glucanosyltransglycosylase	>jgi|TrireRUTC30_1|103899	β-1,3-Glucanosyltransferase
	>jgi|Trias1|152776	β-1,3-Glucanosyltransglycosylase	>jgi|TrireRUTC30_1|113858	β-1,3-Glucanosyltransferase
	>jgi|Trias1|93680	β-1,3-Glucanosyltransglycosylase		
**GH74**	>jgi|Trias1|54925	Xyloglucanase	>jgi|TrireRUTC30_1|111943	Xyloglucanase
**GH79**	>jgi|Trias1|191352	Putative glucuronidase		
**GH92**	>jgi|Trias1|135494	α-1,2-Mannosidase	>jgi|TrireRUTC30_1|94562	α-1,2-Mannosidase
	>jgi|Trias1|159955	Putative α-1,2-mannosidase		
	>jgi|Trias1|24699	α-1,2-Mannosidase		
**GH93**	>jgi|Trias1|41471	Putative exo-α-L-1,5-arabinanase		
**GH95**	>jgi|Trias1|146605	Putative α-fucosidase		

Clear differences in the number and nature of GH proteins secreted by S4F8 and Rut C30 were evident, with S4F8 expressing a larger range of GH families (32 versus 24 GH families in S4F8 and Rut C30, respectively), and more protein representatives per GH family (Figure 3). More proteins belonging to GH families 3 (β-glucosidase/β-xylosidase), 5 (various), 11 (endoxylanase), 16 (transglycosylase and glucanosyltransferase), 28 (polygalacturonase), 31 (α-glucosidase/α-xylosidase), 62 (α-L/N-arabinofuranosidase), 72 (glucanosyltransglycosylase) and 92 (mannosidase) were detected for S4F8. Representatives of GH families 10 (endoxylanase), 12 (endoglucanase), 15 (starch-related), 17 (glucan 1,3-β-glucosidase), 18 (chitinase), 43 (xylosidase), 47 (α-mannosidase), 79 (glucoronidase), 93 (exo-arabinase) and 95 (fucosidase) were unique to S4F8, whereas only representatives of GH families 30 (β-glucocerebrosidase) and 61 (endoglucanases, recently reclassified as copper-dependent lytic monooxygenases in Auxiliary Activity (AA) family 9 of the CAZy database) were unique to Rut C30.

Closer inspection of the secreted proteins detected in the secretomes revealed that in general, an equivalent or higher number of the cellulases (exoglucanase, endoglucanase, and β-glucosidase), main chain hemicellulases (endoxylanase, β-xylosidase), and side chain hemicellulases (for example, α-galactosidase and α-arabinofuranosidase) were secreted by S4F8 (Table 2, Figure 3B; Additional file 4: Figure S1). For example, eight β-glucosidases (representing families GH1 and GH3), five endoxylanases (GH11 and GH10) and three α/β-xylosidases (GH3 and GH43) were identified for S4F8, as opposed to six β-glucosidases (GH1 and GH3), three endoxylanases (GH11), and two α/β-xylosidases (GH3) in Rut C30.

It has been shown that, depending on the substrate, *T. reesei* strains generally produce higher amounts of GH proteins relative to other cellulolytic species such as *Aspergillus fumigatus*, *Fusarium verticilliodes*, *Fusarium graminearum*, and *Phanerochaete chrysosporium*[28,30,49,50]. These typically include two cellobiohydrolases, eight endoglucanases, and seven β-glucosidases [51], of which both the cellobiohydrolases (>jgi|TrireRUTC30_1|125125 and >jgi|TrireRUTC30_1|122470, representing GH6 and GH7), four endoglucanases (>jgi|TrireRUTC30_1|5304, >jgi|TrireRUTC30_1|139633, >jgi|TrireRUTC30_1|72489, and >jgi|TrireRUTC30_1|122518, representing GH5, 7 and 61) and six β-glucosidases (>jgi|TrireRUTC30_1|25095|, >jgi|TrireRUTC30_1|125268, >jgi|TrireRUTC30_1|136547, and >jgi|TrireRUTC30_1|8750, representing GH3, and > jgi|TrireRUTC30_1|127115, and >jgi|TrireRUTC30_1|77989, representing GH1) were detected in the Rut C30 secretome.

As no information on the typical lignocellulolytic enzymes expressed by *T. asperellum* has been described previously, a similar analysis was not possible for strain S4F8. However, it was apparent from the secretome analysis that S4F8 secreted a well-balanced cellulolytic complex in SCB SSF, which included most of the core cellulases typically associated with lignocellulose hydrolysis. This included two cellobiohydrolases (>jgi|Trias1|46985, representing GH7 and >jgi|Trias1|84972, representing GH6), four endoglucanases (>jgi|Trias1|356270, >jgi|Trias1|61451, >jgi|Trias1|57926, and >jgi|Trias1|177701, representing GH5, 7 and 12) and eight β-glucosidases (>jgi|Trias1|128828, >jgi|Trias1|151383, >jgi|Trias1|203210, >jgi|Trias1|23916, >jgi|Trias1|63437, and >jgi|Trias1|65584, representing GH3, and >jgi|Trias1|63798 and >jgi|Trias1|55643, representing GH1).

In general, a diverse spectrum of depolymerization and accessory enzymes were detected in the two fungal secretomes, which agrees with the consensus that more complex substrates, such as untreated SCB, will lead to the induction of more complex lignocellulolytic cocktails. The lignocellulosic enzyme profile secreted by fungi is known to be dependent on the type and composition of the carbon source used, and it is to be expected that the S4F8 and Rut C30 secretomes will vary if carbon sources other than SCB are used, as was recently shown in a *Penicillium echinulatum* secretome study [10]. Compared with the *P. echinulatum* secretome on SCB, which contained predominantly cellulolytic enzymes [10], both S4F8 and Rut C30 produced a more diverse GH profile, with a higher number of β-glucosidases and hemicellulases (both main and side chain) detected during SSF on SCB.

Interestingly, most of the hydrolytic activities proposed by a recent hierarchical model for sugarcane cell wall degradation [52] were detected in this study. According to that model, hydrolysis of the cell walls of untreated sugarcane require initial attack by pectinases (endo-polygalacturonase, pectin-methyl-esterase, α-arabinofuranosidase, and β-galactosidase), together with 1,3-1,4-β-D-glucanases to hydrolyse β-glucans. To this end, three exo-/endo-polygalacturonases (GH28: >jgi|Trias1|204961, >jgi|Trias1|74014, and >jgi|TrireRUTC30_1|133383) were detected in the in the S4F8 and Rut C30 secretomes, while one pectin-methyl-esterase (carbohydrate esterase family 8 (CE8): >jgi|Trias1|82670) was detected in the S4F8 secretome only. Various α-arabinofuranosidases (GH54, GH62) and β-galactosidases (GH35) relevant to pectin degradation were also detected in the S4F8 and Rut C30 secretomes.

Proteomic analysis of secretomes can also shed light on the relative production or secretion levels of a given protein as measured by its abundance (that is, how many times a given protein is detected). The relative abundance of the GH proteins in the respective secretomes (expressed as fold increase relative to the other strain) (Table 3) indicated that seven glycoside hydrolases, including α-D-galactosidase (GH27), α-1,2-mannosidase (GH92), β-mannosidase (GH2), endo-1,4-β-xylanase (GH11), β-N-acetylhexosaminidase (GH20), and *N, O*-diacetylmuramidase (GH25), were significantly more abundant in S4F8 than in Rut C30, whereas an α-D-galactosidase (GH27) and β-glucosidase (GH1) were significantly more abundant in the Rut C30 secretome.

**Table 3:** **Summary of protein abundance differences detected for glycoside hydrolase (GH) proteins common to the *Trichoderma asperellum *** **S4F8 and *Trichoderma reesei *** **Rut C30 secretomes**

**Protein IDs**	**SignalP**	**Biological function**	**Fold increase**
**Increased abundance in *T. aperellum *** **S4F8**			
>jgi|Trias1|83211 / >jgi|TrireRUTC30_1|134945	Yes	Endo-1,4-β-xylanase 1 (GH11)	2.6
>jgi|Trias1|152923 / >jgi|TrireRUTC30_1|12549	Yes	β-Mannosidase (GH2)	3.1
>jgi|Trias1|157721 / >jgi|TrireRUTC30_1|99285	Yes	β-N-acetylhexosaminidase (GH20)	3.6
>jgi|Trias1|24131 / >jgi|TrireRUTC30_1|13308	Yes	*N*,*O*-diacetylmuramidase (GH25)	3.7
>jgi|Trias1|59499 / >jgi|TrireRUTC30_1|6433	Yes	α-D-galactosidase (GH27)	3.8
>jgi|Trias1|127630 / >jgi|TrireRUTC30_1|25104	Yes	Glucan 1,3-β-glucosidase (GH55)	2.9
>jgi|Trias1|135494 / >jgi|TrireRUTC30_1|94562	Yes	α-1,2-Mannosidase (GH92)	3.4
**Increased abundance in *T. reesei *** **Rut C30**			
>jgi|TrireRUTC30_1|127115 / >jgi|TrireRUTC30_1|77989 / >jgi|Trias1|55643	No	β-Glucosidase (GH1)	3.6
>jgi|TrireRUTC30_1|71638 / >jgi|Trias1|47755	Yes	α-D-Galactosidase (GH27)	3.2

## Conclusion

The hyperproducing and hypersecreting *Trichoderma reesei* Rut C30 mutant strain is considered a paradigm among cellulase-producing *T. reesei* strains and has served as the benchmark for industrial cellulase production. However, driven by an increased demand for cheaper and more efficient lignocellulolytic enzyme cocktails, considerable research effort is focused on the further improvement of the ‘lignocellulose degradome’ of *T. reesei* and in finding alternative enzymes that could potentially replace and/or supplement *T. reesei* cocktails to overcome the remaining challenges for commercially feasible biomass-to-ethanol conversion processes. The results presented here indicate that *T. asperellum* strain S4F8, which grew particularly well on SCB, produced a lignocellulolytic cocktail in an SSF process with hemicellulase and β-glucosidase abilities that exceeded those of *T. reesei* Rut C30. We provide the first comprehensive secretome analysis for a *T. asperellum* strain, and reveal that its secretome contains a more complex cocktail of GH family representatives than *T. reesei* Rut C30. Furthermore, the efficacy of untreated SCB in an SSF process highlights the suitability of this cheap, widely available agroindustrial waste product as a substrate for the production of fungal lignocellulolytic enzymes. In summary, the *T. asperellum* strain S4F8 has significant potential for the production of lignocellulolytic enzymes, and merits further investigation, which could include in-depth characterization of individual enzymes or multi-enzyme complexes, the evaluation of other lignocellulosic substrates, optimization of the SSF culture conditions, and strain improvement.

## Methods

### Strains, media, and chemicals

The *T. reesei* Rut C30 (ATCC 56765) strain [53] was obtained from the culture collection of the Department of Microbiology, Stellenbosch University, South Africa. The S4F8 strain was isolated from a forest soil sample collected from the Oribi Gorge, KwaZulu-Natal, South Africa.

Strains were maintained on malt extract agar (MEA; Sigma Aldrich, Seelze, Germany) or potato dextrose agar (PDA; Merck KGaA, Darmstadt, Germany) at 30°C and stored on MEA slants at room temperature. When required, strains were cultured in yeast peptone dextrose (YPD) broth (Merck, KGaA). All chemicals, media components, and supplements were analytical grade.

### Isolation of lignocellulolytic fungi

To select for fungi capable of growth on cellulosic substrates, 1 g soil sample was resuspended in 10 ml physiological salt solution (8.5 g/l NaCl), and plated onto agar plates containing synthetic medium (1.76 g/l yeast nitrogen base, 5 g/l ammonium sulfate) with either 10 g/l HEC or CMC as sole carbon source. Degradation of amorphous cellulose was confirmed by the presence of clear halos around the colonies following Congo Red staining [54].

### Molecular identification

Isolate S4F8 was inoculated at 10^4^ spores/ml into YPD broth and incubated for 5 days at 30°C with constant agitation (100 rpm). Total genomic DNA was isolated using the ZR Fungal/Bacterial DNA Miniprep^TM^ kit (Zymo Research Corp., Orange, CA, USA). Amplification of the ITS regions (ITS1 and 2) of the nuclear ribosomal RNA gene was performed using primers ITS1 (5′-TCCGTAGGTGAACCTTGCGG-3′) and ITS4 (5′-TCCTCCGCTTATTGATATGC-3′). with total genomic DNA as template [55].

The 25 μl PCR reaction mix contained approximately 100 ng genomic DNA, 0.2 μmol/l of each primer, 10 μmol/l deoxynucleotides, and 1 U ExTaq (TaKara Bio Inc., Otsu Shiga, Japan). The PCR reaction was carried out in a GeneAmp PCR System 2400 (Perkin Elmer), using 30 cycles of denaturation at 94°C for 1 minute, annealing at 58°C for 1 minute, and extension at 72°C for 1 minute, with a final extension step at 72° for 7 minutes. PCR products were visualized by electrophoresis in 0.8% (w/v) agarose (Sigma Aldrich) gels at 80 V and the approximately 600 bp amplicon was excised and gel-purified using the Zymoclean^TM^ Gel DNA Recovery Kit (Zymo Research Corp.). The fragment was cloned using the InsTAclone^TM^ PCR Cloning Kit (Fermentas, Maryland, USA) and transformed into *Escherichia coli* DH5α.

Sequencing of triplicate clones was carried out with an Applied Biosystems 3130xl Genetic Analyzer (Central Analytical Facility, Stellenbosch, South Africa). Sequence alignment and analysis were performed with DNAMAN software (Lynnon Corporation, Canada) and the final consensus sequence subjected to a similarity search using the BLASTn algorithm (http://blast.ncbi.nlm.nih.gov). The TrichOKey2 oligonucleotide DNA BarCode system [31] and TrichoBLAST (http://www.isth.info/tools/blast/index.php) were used for final identification.

### Solid-state fermentation

**Figure 4:**
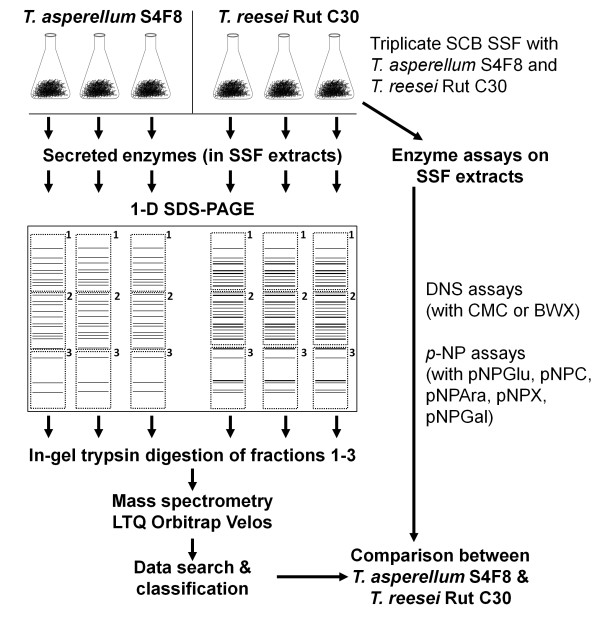
**Schematic representation of the experimental design used to compare extracellular proteins of *Trichoderma asperellum *** **S4F8 and *Trichoderma reesei *** **Rut C30 on sugarcane bagasse (SCB) during solid-state fermentation (SSF).**

A flow diagram for the cultivation of the fungi, enzyme assays, and proteomic analysis is shown in Figure 4. The fungal strains were cultured on MEA, and allowed to sporulate. A quantity (5 g) of dry, untreated SCB (TSB Sugar RSA, Mpumalanga, South Africa) was dispensed into 250 ml Erlenmeyer flasks, 10 ml of a mineral salt solution (6 g/l Na_2_HPO_4_, 3 g/l NaH_2_PO_4_, 1 g/l KCl, and 0.1 g/l MgSO_4_^.^7H_2_O, adjusted to pH 7.0 with concentrated HCl) was added, and the mixture was sterilized by autoclaving for 15 minutes at 121°C.

For enzyme activity profiles and secretome analysis, suspensions of *T. reesei* Rut C30 and *T. asperellum* S4F8 spores in physiological salt solution were inoculated in triplicate onto sterile SCB at approximately 2 × 10^7^ spores per gds. After 3 days of incubation under standard SSF conditions (30°C in darkness without humidity control; culture condition A), 100 ml of 0.05 mol/l citrate-phosphate buffer (pH 7.0) was added to the flasks and incubated with the bagasse/fungus mixture for 30 minutes with agitation at 200 rpm [56]. The supernatant containing the secretome extracts was filtered through several layers of Miracloth (Merck) and either used directly for enzyme assays, or lyophilized (Virtis Freeze Dryer 6 K) for secretome analysis. Modifications to the standard SSF culture conditions to optimize lignocellulosic enzyme production included incubating SSF cultures in constant fluorescent light (culture condition B), in darkness at a controlled RH of 90% using a Hotpack CO_2_ incubator (culture condition C), or in darkness at 26°C (culture condition D).

### Enzyme assays

Endoglucanase and endoxylanase activities were quantified using a scaled-down dinitrosalicyclic acid (DNS) assay with 10 g/l low-viscosity CMC and BWX, respectively, in 0.05 mol/l citrate buffer pH 5.0 at 50°C [57,58]. The β-glucosidase, cellobiohydrolase I, α-arabinofuranosidase, β-xylosidase and α-galactosidase activities were determined with the respective *p*-nitrophenyl-D-β-glucopyranoside (pNPGlu), *p*-nitrophenyl-D-β-cellobiose (pNPC), *p*-nitrophenyl-L-α-arabinofuranoside (pNPAra), *p*-nitrophenyl-β-D-xylopyranoside (pNPX) and *p*-nitrophenyl-α-D-galactoside (pNPGal) substrates. The corresponding standard curves were prepared with 0.2-10 μmol/l glucose or xylose, and 0.4 to 0.8 mg/ml *p*-nitrophenyl. Enzyme activities are represented as the mean values of triplicate experiments, and expressed in units per gram dry SCB, with one unit defined as the amount of enzyme required to release 1 μmol of product per minute from the appropriate substrate under assay conditions. Statistical inferences were calculated using one way ANOVA (SigmaPlot version 11; Systat Software Inc., Germany).

### SDS-PAGE analysis

Lyophilized secretome extracts were reconstituted with deionized water, protein concentrations were determined with the detergent compatible (DC) Lowry protein assay kit (BioRad, Melville, NY, USA), and 20 μg samples were loaded in triplicate into a 10% SDS-polyacrylamide gel for electrophoresis. Protein profiles were visualized by overnight staining with 5 g/l Coomassie Blue G-250 (Merck), followed by destaining with 100 ml/l acetic acid [59].

### In-gel trypsin digestion

Triplicate lanes from the SDS-PAGE gel were divided into three fractions for analysis by MS. Each fraction was diced into smaller pieces (1 mm × 1 mm) to simplify subsequent sample preparation. The collection of smaller pieces from each fraction was washed twice with water followed by 50% (v/v) acetonitrile for 10 minutes. The acetonitrile was replaced with 50 mmol/l ammonium bicarbonate and the pieces incubated for 10 minutes; this was repeated two more times. All the gel pieces were then incubated in 100% acetonitrile until they turned white, after which they were vacuum-dried. Proteins were reduced with 10 mmol/l DTT for 1 hour at 57°C. This was followed by brief washing steps with 50 mmol/l ammonium bicarbonate followed by 50% acetonitrile, before proteins were alkylated with 55 mmol/l iodoacetamide for 1 hour in the dark. The gel pieces were washed with 50 mmol/l ammonium bicarbonate for 10 minutes, followed by 50% acetonitrile for 20 minutes, before being vacuum-dried*.* The gel pieces were digested with 100 μl of a 10 ng/μl trypsin solution at 37°C overnight. The resulting peptides were extracted twice with 70% acetonitrile in 0.1% formic acid for 30 minutes followed by 100% acetonitrile for 30 minutes. The resulting peptides were desalted using Stage tips [60]. Dried peptides from each fraction were dissolved in 5% acetonitrile in 0.1% formic acid, from which 10 μl injections were prepared for nano-LC chromatography.

### Mass spectrometry

All experiments were performed on a Thermo Scientific EASY-nLC II connected to a LTQ Orbitrap Velos Mass Spectrometer (Thermo Scientific, Bremen, Germany) equipped with a nano-electrospray source. For liquid chromatography, separation was performed on an EASY Column (2 cm, ID 100 μm, 5 μm, C18) pre-column, followed by a XBridge BEH130 NanoEase column (15 cm, ID 75 μm, 3.5 μm, C18) with a flow rate of 300 nl/min. The gradient used was 5 to 17% B in 5 minutes, 17 to 25% B in 90 minute, 25 to 60% B in 10 minutes, 60 to 80% B in 5 minutes, and kept at 80% B for 10 minutes. Solvent A was aqueous solution in 0.1% formic acid, and solvent B was 100% acetonitrile in 0.1% formic acid.

The mass spectrometer was operated in data-dependent mode to automatically switch between Orbitrap-MS and LTQ-MS/MS acquisition. Data were acquired using the Xcalibur software package. The precursor ion scan MS spectra (*m/z* 400 to 2000) were acquired in the Orbitrap with resolution R = 60 000 with 1 × 10^6^ accumulated ions. The 20 most intense ions were isolated and fragmented in a linear ion trap (1.5 × 10^4^ accumulated ions) using collision-induced dissociation. The lock mass option (polydimethylcyclosiloxane; *m/z* 445.120025) enabled accurate mass measurement in both the MS and MS/MS modes. In data-dependent LC-MS/MS experiments, dynamic exclusion was used with an exclusion duration of 60 seconds. MS conditions were 1.8 kV with a capillary temperature of 250°C, and no sheath and auxiliary gas flow. For MS/MS, the ion selection threshold was 500 counts, activation Q-value was 0.25 and activation time was 10 milliseconds.

Eighteen raw files were processed using MaxQuant 1.2.2.5 [61] for protein identification and label-free quantification, using the Joint Genome Institute (JGI) database for *Trichoderma asperellum* CBS 433.97 version 1.0 (http://genome.jgi.doe.gov/Trias1/Trias1.home.html) and *Trichoderma reesei* RUT C-30 version 1.0. (http://genome.jgi.doe.gov/TrireRUTC30_1/TrireRUTC30_1.home.html). Carbamidomethyl cysteine was set as the fixed modification, with oxidized methionine, acetylation (N-term), deamidation (NQ) and Pyr-Q (Gln to 2-pyrrolidone-5-carboxylic acid-Glu) and Pyr-E (Glu to 2-pyrrolidone-5-carboxylic acid-Glu) as the variable modification. The precursor mass tolerance was set to 20 ppm, and the fragment mass tolerance to 0.8 Da. Two missed tryptic cleavages were allowed, with a minimal peptide length of six amino acids. Proteins that were identified were reported as single groups. Only proteins containing at least one unique peptide were considered. The criteria that were applied included a peptide and protein FDR of 1% (0.01), and a posterior error probability of 0.01. These extremely strict parameters guaranteed that proteins would be identified with high confidence.

Proteins that were differentially expressed between *T. asperellum* S4F8 and *T. reesei* Rut C30 were determined using Maxquant LFQ intensity values as a parameter for protein abundance [62]. Subsequent statistical analysis was performed using Perseus. Proteins with a fold regulation of at least two and *P*-value of at least 0.05 were accepted. Proteins identified in only one species were required to be identified with at least two unique peptides to ensure abundance differences were real and not due to non-identification of parent ions by the MS analysis. SignalP (http://www.cbs.dtu.dk/services/SignalP) was used to identify possible secretion signals.

### Proteome network analysis

Each secreted protein in the Rut C30 and S4F8 secretomes was annotated according to broad functional categories and their specific enzymatic activity or molecular function (see Additional file 3: Table S3). In addition, proteins known to be members of a specific GH family were annotated as such. A custom-built Perl program was written in order to create a network in which the broad functional categories and the proteins were nodes and edges were created between the categories and the proteins assigned to them. The program also created a second network, in which the GH families and the proteins were nodes, and edges were created between GH family nodes and the proteins assigned to them. The union of these two networks was taken and a complete breadth-first search performed, starting from all GH family nodes. The nodes and edges selected by the breadth-first search were used to create a new network, which was visualized with Cytoscape [63]. A spring-embedded layout was used on the network and nodes were further manually arranged for better visualization. A Perl program was also used to create network annotations in order to control both node label size and node colour (purple if from *T. reesei* Rut C30, blue if from *T. asperellum* S4F8. and red if the protein was found in both secretomes). Node label positions were further adjusted manually.

## Abbreviations

AA: Auxiliary activity; ATCC: American type culture collection; BLAST: Basic local alignment search tool; BWX: Beechwood xylan; CMC: Carboxymethylcellulose; DNS: Dinitrosalicyclic acid; DTT: Dithiothreitol; FDR: False discovery rate; FPU: Filter paper units; gds: Gram of dry substrate; GH: Glycoside hydrolase; HEC: Hydroxyethylcellulose; ITS: Internal transcribed spacer; JGI: Joint Genome Institute; LC-MS: Liquid chromatography-mass spectrometry; MEA: Malt extract agar; MS/MS: Tandem mass spectrometry; PCR: Polymerase chain reaction; PDA: Potato dextrose agar; SCB: Sugarcane bagasse; SDS-PAGE: Sodium dodecyl sulfate–polyacrylamide gel electrophoresis; SSF: Solid-state fermentation; pNPAra: *p*-nitrophenyl-L-α-arabinofuranoside; pNPC: *p*-nitrophenyl-D-β-cellobiose; pNPGal: *p*-nitrophenyl-α-D-galactoside; pNPGlu: *p*-nitrophenyl-D-β-glucopyranoside; pNPX: *p*-nitrophenyl-β-D-xylopyranoside; RH: Relative humidity; YPD: Yeast peptone dextrose.

## Competing interests

The authors declare that they have no competing interests.

## Authors’ contributions

IJM contributed to the isolation and identification of *T. asperellum* S4F8, the initial SSF studies, enzyme activity profiling, functional characterization of proteomic data, and drafting of the manuscript. NVW conceptualized this study and contributed to the optimization of the SSF, enzyme activity profiling, protein SDS-PAGE analysis, quantification of enzyme activities, and drafting of the manuscript. SS contributed to the peptide mass spectrometry, protein data, and statistical analysis. DJ contributed to proteome network analysis. MVB contributed to data analysis and manuscript revision. HV contributed to the design and coordination of the study, and drafting and revision of the manuscript. All authors read and approved the final manuscript.

## Supplementary Material

Additional file 1: Table S1 All peptides detected with posterior error probability (PEP) < 0.01 and at least one unique peptide in secretomes of *Trichoderma asperellum* S4F8 and *Trichoderma reesei* Rut C30 grown in sugarcane bagasse (SCB) solid-state fermentation (SSF). Raw data from liquid chromatography tandem mass spectrometry analysis. Click here for file

Additional file 2: Table S2 All the proteins detected with posterior error probability (PEP) < 0.01 in SCB SSF secretomes of *Trichoderma asperellum* S4F8 and *Trichoderma reesei* Rut C30 grown in sugarcane bagasse (SCB) solid-state fermentation (SSF). Raw data from liquid chromatography tandem mass spectrometry analysis. Click here for file

Additional file 3: Table S3 Grouping of secreted proteins for the SCB SSF secretomes of *Trichoderma asperellum* S4F8 and *Trichoderma reesei* Rut C30 for functional annotation network analysis. Raw data from functional annotation network analysis. Click here for file

Additional file 4: Figure S1 Functional annotation network analysis of proteins involved in cellulose, hemicellulose, pectin, chitin, and starch degradation, cell wall biosynthesis and morphogenesis and general carbohydrate transport and metabolism detected in the *Trichoderma asperellum* S4F8 and *Trichoderma reesei* Rut C30 secretomes. Functional annotation network analysis with enzyme identities included. Click here for file
