# Supplementary figures and images for: Comparative secretome analysis of Trichoderma asperellum S4F8 and Trichoderma reesei Rut C30 during solid-state fermentation on sugarcane bagasse
Source: Biotechnol Biofuels. 2013 Nov 29;6:172. doi: 10.1186/1754-6834-6-172 (PMC4177139; doi:10.1186/1754-6834-6-172)

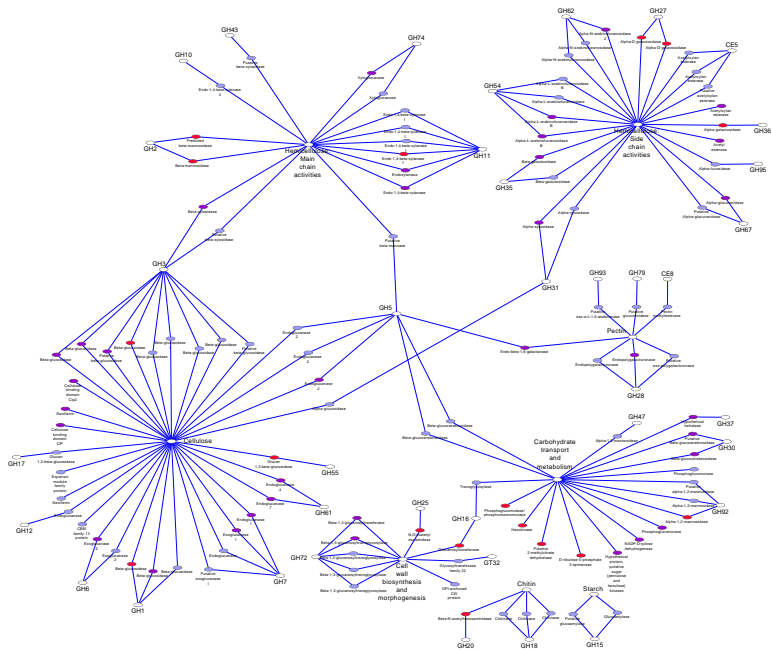

Supplement: Additional file 4: Figure S1 — Functional annotation network analysis of proteins involved in cellulose, hemicellulose, pectin, chitin, and starch degradation, cell wall biosynthesis and morphogenesis and general carbohydrate transport and metabolism detected in the Trichoderma asperellum S4F8 and Trichoderma reesei Rut C30 secretomes. Functional annotation network analysis with enzyme identities included. [file 1754-6834-6-172-S4.pdf]
